# Zinc Status and Risk of Cardiovascular Diseases and Type 2 Diabetes Mellitus—A Systematic Review of Prospective Cohort Studies

**DOI:** 10.3390/nu8110707

**Published:** 2016-11-05

**Authors:** Anna Chu, Meika Foster, Samir Samman

**Affiliations:** 1 Department of Human Nutrition, University of Otago, Dunedin 9054, New Zealand; anna.chu@otago.ac.nz (A.C.); meika.foster@otago.ac.nz (M.F.); 2 Discipline of Nutrition and Metabolism, School of Life and Environmental Sciences, University of Sydney, Sydney 2006, NSW, Australia

**Keywords:** zinc, cardiovascular diseases, diabetes, epidemiology

## Abstract

Zinc is an essential trace element with proposed therapeutic effects in Type 2 diabetes mellitus (DM), however, the associations between zinc status and the prospective risks of cardiovascular diseases (CVD) and Type 2 DM have not been evaluated. The current systematic review aims to determine the relationships between zinc intake or plasma/serum zinc levels and prospective incidence of CVD and Type 2 DM. Fourteen papers describing prospective cohort studies were included, reporting either CVD (*n* = 91,708) and/or Type 2 DM (*n* = 334,387) outcomes. Primary analyses from four out of five studies reported no association between zinc intake and CVD events, when adjusted for multiple variables. Higher serum zinc level was associated with lower risk of CVD in three out of five studies; pronounced effects were observed in vulnerable populations, specifically those with Type 2 DM and patients referred to coronary angiography. The limited evidence available suggests no association between zinc status and Type 2 DM risk. Further investigations into the mechanisms of zinc’s action on the pathogenesis of chronic diseases and additional evidence from observational studies are required to establish a recommendation for dietary zinc in relation to the prevention of CVD and Type 2 DM.

## 1. Introduction

Chronic metabolic diseases, such as cardiovascular diseases (CVD) and Type 2 diabetes mellitus (DM), represent a significant proportion of non-communicable disease (NCD) deaths around the world [1]. In 2012, 17.5 million and 1.5 million deaths were attributed to CVD and Type 2 DM, respectively. Deaths due to NCD are projected to increase, with the majority occurring in low- and middle-income countries (LMIC). The role of diet and physical activity in the prevention and management of chronic metabolic diseases is recognised by numerous health organisations [2,3]. While there is substantial evidence for optimal dietary patterns and macronutrient compositions for the prevention of diseases, the influence of micronutrient intakes, such as zinc, on the risk of developing chronic metabolic diseases remains to be elucidated. 

Zinc is an essential trace element with antioxidant activity and functions related to energy metabolism and growth [4]. In addition, zinc has been shown to serve a regulatory role in many signalling pathways, including potentiation of leptin and insulin signalling [5]. The involvement of zinc in immunity has been demonstrated consistently in cellular studies [6]; specifically, a protective role of zinc was shown for autoimmune diseases, including multiple sclerosis [7] and Type 1 DM [8]. Recent genome-wide association studies identified the potential roles of Zinc Transporter-8 (ZnT8; SLC30A8) and zinc status in the pathogenesis and management of Type 2 DM [9]. Meta-analyses of randomised controlled trials (RCT) involving patients with Type 2 DM revealed improvements in measures of glycaemic control [10] and dyslipidaemia [11] following zinc supplementation. The mechanisms of action have been proposed to involve improvements in the stability of insulin within pancreatic β-cells and insulin sensitivity in peripheral tissues. While the therapeutic effects of zinc in Type 2 DM have been proposed [10,11], the associations between zinc status and the prospective risks of developing CVD and Type 2 DM have not been evaluated. 

Findings in cell and animal studies support the protective role of zinc against the risk factors of CVD, such as the development of atherosclerosis. The modulating effects of zinc on the formation of atherosclerotic plaque are primarily attributed to the role of zinc in supporting the structural integrity of endothelial cells and attenuating lipid peroxidation through zinc-regulated redox signalling pathways [5,12]. In cultured endothelial cells, the aberrant expression of inducible nitric oxide synthase (iNOS) forms the basis of endothelial dysfunction and pathogenesis of atherosclerosis. Zinc has been shown to regulate the activity of iNOS, reversing the adverse effects of inflammation on the endothelium [13]. In addition, zinc supplementation has been shown to reduce atheroma formation and plasma and arterial wall lipid peroxidation in rabbits fed a high cholesterol diet [14]. Taken together, the growing evidence derived from experimental models provides biological plausibility for a potential role of zinc in CVD prevention. 

Furthermore, the association between zinc deficiency, low grade inflammation and immune response supports the importance of optimal zinc status in modulating the inflammatory events required for atherosclerosis development in humans [15,16]. While RCT designed to study the effects of zinc supplementation on the development of CVD are limited, a number of observational studies have explored the relationship between zinc status and CVD outcomes [17,18]. In a Finnish nested case-control study, the highest tertile of serum zinc concentration was associated with 43% reduction in the relative risk (RR) of CVD death compared to those in the lowest tertile of serum zinc [17]. Similar trends were observed in an urban Indian population, where higher dietary zinc intakes and higher concentrations of serum zinc concentrations were correlated with reduced prevalence of coronary artery disease and Type 2 DM [18].

To date, no systematic review has explored the link between zinc status and the risk of developing chronic metabolic diseases, specifically Type 2 DM and CVD. Therefore, our aim is to determine the relationship between zinc intake, plasma/serum zinc concentrations and prospective incidence of CVD and Type 2 DM in cohort studies.

## 2. Methods

### 2.1. Search Strategy

**Figure 1:**
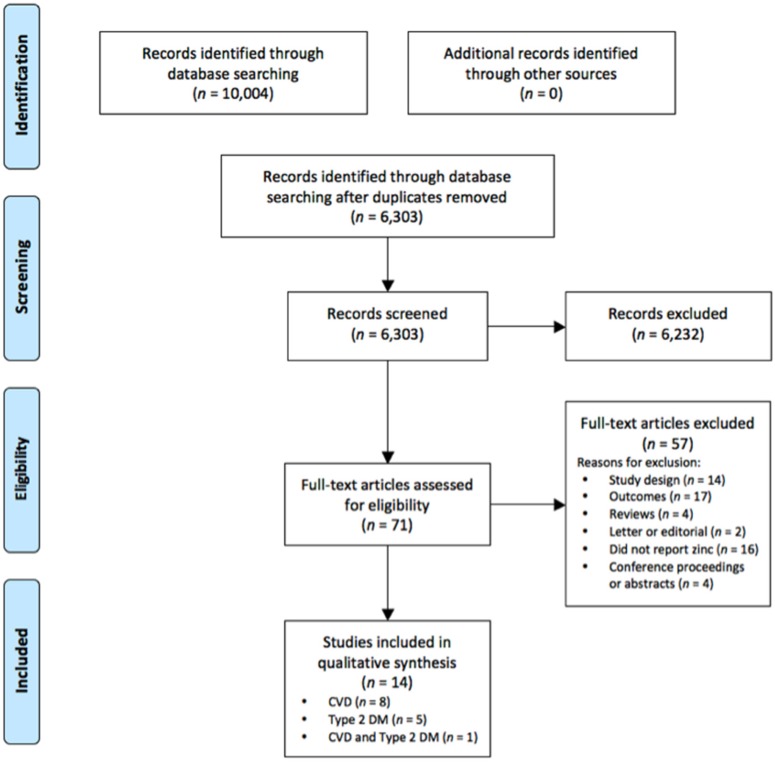
PRISMA diagram showing the systematic review process.

A literature search was conducted of PubMed, Cochrane Central Register of Controlled Trials, EMBASE, MEDLINE and Web of Science from inception to 28 June 2016. Search terms used were zinc AND [(dietary OR supplement*) OR (plasma OR serum)] AND (diabetes OR cardiovascular diseases OR metabolic syndrome OR cerebrovascular diseases OR chronic kidney disease). Related terms and MeSH terms were used where available. Studies were limited to humans and English language. Full search details for the search of “zinc and diabetes” in the EMBASE database are presented in Table S1 (Supplemental Materials). Figure 1 shows the Preferred Reporting Items for Systematic reviews and Meta-Analyses (PRISMA) flowchart describing the electronic search outcomes and selection process [19]. Review questions, search strategies and inclusion criteria were prospectively specified and registered with PROSPERO register at http://www.crd.york.ac.uk/PROSPERO/(CRD42015020589). 

### 2.2. Study Eligibilty Criteria

Prospective cohort studies that examined the association between zinc status and the risk of developing chronic metabolic diseases (Type 2 DM and CVD) were eligible for inclusion. The definitions of diseases derive from the International Classification of Diseases [20]; for instance, CVD encompasses coronary heart disease and cerebrovascular disease. Apparently healthy participants and those with existing co-morbidities were included in this review. Studies were selected if plasma/serum zinc concentration or zinc intake (dietary and/or supplemental) were measured as indices of zinc status, in conjunction with exposure risk for developing chronic diseases stratified by zinc status. Plasma and serum zinc concentrations were grouped to represent systemic zinc concentration; the terms plasma and serum are used interchangeably in this report to reflect the measurement provided by the study authors. All other studies and review papers on the topic were excluded. Two independent reviewers screened the title and abstract of each citation identified in the literature search to determine the paper’s eligibility for full review. The full report of selected citations were retrieved and screened by two investigators independently. 

### 2.3. Data Extraction and Quality Assessment of Included Studies

Data from all included studies were extracted independently by two investigators and any differences were resolved by discussion. The data extraction sheet included participants’ characteristics (age, sex, disease characteristics), number of included participants and cases identified, zinc biomarker used and method of analysis, years of follow up, and characteristics of outcome measures (risk estimates and precisions, statistical models used, adjustments for confounding measures). Where available, outcome measures for CVD events were extracted as fatal, non-fatal and combined events. For studies where multiple models were presented with different statistical adjustments, outcome data of the unadjusted and fully adjusted models were extracted, along with the identified covariates. Only published data were included as we did not make contact with authors of included studies. Meta-analyses for the relationships were deemed inappropriate due to the variability in statistical methods and reporting of the outcome statistics.

### 2.4. Quality Assessment

All individual studies were assessed for their risk of biases that are specific to observational studies as recommended by the Grading of Recommendations Assessment, Development and Evaluation (GRADE) guidelines [21]. Two independent reviewers assessed the potential risk of biases, including failure to develop and apply appropriate eligibility criteria, flawed measurement of exposure and/or outcome, failure to adequately control for confounding variables and incomplete or inadequately short follow up. Data on risk of bias in individual studies were entered into Review Manager 5.3 [22]. 

The adequacy of controlling for confounding variables was scored by a defined checklist of confounding variables relevant to the specific exposure measure: zinc intake or serum zinc concentration. For statistical models using zinc intake as an exposure, eight types of variables were considered to be important confounding factors for consideration, giving a total score of 5, as follows. Where models included an aspect of family history, comorbidities, lifestyle factors, anthropometry, dietary factors or medications, the study was given a score of 0.5 for each variable (maximum score of 3). Additionally, saturated fat and *trans* fat intakes were considered to be essential dietary factors for the zinc intake models involving CVD as an outcome, and therefore studies that included these variables in the fully adjusted statistical models were given a score of 1 for each variable. For zinc intake models with Type 2 DM as an outcome, saturated fat and dietary fibre intakes were considered to be significant confounding factors and therefore inclusion of each factor was given a score of 1. 

In the statistical models considering serum zinc concentration as an exposure factor and CVD as an outcome, the essential confounding variables (each representing a score of 1) were as follows: total cholesterol or low density lipoprotein-cholesterol (LDL-C) concentrations; high density lipoprotein-cholesterol (HDL-C) concentration; and a biomarker of inflammation, giving a total score of 3. For serum zinc models with Type 2 DM as an outcome, high density lipoprotein-cholesterol (HDL-C) concentration (score of 0.5) and a biomarker of inflammation (score of 1) were considered to be important confounding variables, along with glycaemic outcomes (fasting glucose or HbA1c) and blood triglycerides, which were given respective scores of 1 and 0.5 (total score of 3). 

In the evaluation of the quality of studies, for those with the highest potential total score of 5, scores less than 2 were considered to be at high risk of bias; scores between 2 and 4 were considered to be at unclear risk of bias; and scores greater than 4 were considered to be at low risk of bias. For those with a highest potential total score of 3, scores less than 1.5 were considered to be at high risk of bias; scores between 1.5 and 2.5 were at unclear risk of bias; and a score of 3 was considered to be at low risk of bias. The factors and associated scores are shown in Table S2 (Supplemental Materials). 

## 3. Results

The electronic database searches identified a total of 6303 citations once duplicates were removed. After screening the titles and abstracts, 6232 citations were excluded as irrelevant to the current review, leaving 71 full texts that were retrieved for eligibility assessment. Of the full texts that were retrieved, 14 papers satisfied the inclusion criteria. Details of study selection and reasons for full text exclusion are presented in Figure 1. 

### 3.1. Zinc Status and CVD Outcomes

Nine papers [23,24,25,26,27,28,29,30,31] describe the relationship between zinc status and fatal and non-fatal CVD events. Two of the identified papers [26,29] report on different aspects of the same study and therefore both were included in the current systematic review. All studies were conducted in high-income countries: USA [24,26,29,30], Britain [25], France [27], Finland [23,28] and Germany [31]. The majority of included participants were recruited from the general population and were apparently healthy at baseline, with the exception of the study conducted by Soinio and colleagues, who recruited patients with Type 2 DM [23]. The number of participants (*n* = 91,708) in the included studies ranged from *n* = 344 to 39,633, with a median of 3655 participants. Five studies [23,25,28,30,31] sampled a mixed-sex population, while two studies reported on males only [24,27] and another study included an exclusively female population [26,29]. Included participants were followed for a mean of 12.4 years (median: 13 years; range: 6.2–19 years). 

The exposure measurement, zinc status, was described as zinc intake (dietary and/or supplemental) [24,25,26,29,30] or serum zinc levels [23,25,27,28,31]. Dietary zinc intakes were derived from food frequency questionnaires (FFQ) for all studies, with the exception of the British National Diet and Nutrition Survey [25], which used a 4-day weighed food record. Questions regarding specific supplements of zinc were used for participants in two of the studies [24,29]. Zinc concentrations in serum or plasma were determined by atomic absorption spectroscopy (AAS) [23,27,28] or colorimetric assays [25,32]. To determine the relationship between measures of zinc status and CVD events, the majority of included articles used multivariate models with stratified levels of zinc intake or serum zinc concentration and adjustments for potential confounding factors. 

Five included papers explored the relationship between zinc intake (dietary and/or supplemental) and CVD events (Table 1). Two studies stratified zinc intake into quintiles [24,30], while another study presented quartiles of zinc intake with an additional stratification at the median of the highest quartile [26]. One study analysed the risk of CVD using standardised zinc intake as a continuous variable [25]. The only study that examined zinc supplement use and CVD risk categorised the study population as groups of users and non-users [29]. Primary analyses from four out of five studies reported no significant association between zinc intake and CVD events, when adjusted for multiple variables [24,26,29,30]. In the remaining study, a significant decrease in vascular disease mortality of 16% was observed per standard deviation (SD) increase in dietary zinc intake (hazard ratio 0.84, CI 95% 0.71, 0.99; *p* = 0.04), after statistical adjustments for age and sex [25]. In secondary analyses, Otto et al. reported greater risk of CVD with increased dietary zinc intake derived from red meat (*p* < 0.01) [30]; however, CVD risk was not associated with total dietary zinc intake from other sources. 

The association between serum zinc concentrations and risk of CVD events was reported in five studies (Table 2). Four studies stratified serum zinc levels into tertiles [28] or quartiles [23,27,31]. In a multivariate model, each quartile decrease of serum zinc concentrations was associated with 10% increased risk of CVD mortality in patients referred for coronary angiography (*p* = 0.038) [31]. A similar effect was noted in patients with Type 2 DM; participants in the lowest quartile of serum zinc level (<14.1 μmol/L) experienced a 37% increased incidence in myocardial infarction (MI) [23]. No significant relationships were observed in two of the five included studies [27,28]. Another study determined the risk of CVD by the continuous variable of standardised plasma zinc levels [25]; while reduced risk of vascular death was observed with higher plasma zinc concentration within the age and sex adjusted statistical model, the association was no longer significant once adjusted for other variables, such as serum cholesterol and lifestyle characteristics [25].

### 3.2. Zinc Status and Type 2 DM

Six studies investigated the relationship between zinc status and the risk of developing Type 2 DM (Table 3 and Table 4). Four of the included studies stratified zinc intake of the study population into quintiles [30,32,33,34], while another study categorised the population into zinc supplement users or non-users [35]. One study included serum zinc levels as a measure of zinc status [36]. The sample size of the six studies ranged from *n* = 2220 to 232,007, with participants followed for 4.8–24 years. All included participants were recruited by population-based methods and were apparently healthy at baseline. 

At the 24-year follow up of the Nurses’ Health Survey, increased total zinc intake was associated with lower Type 2 DM risk, after multiple statistical adjustments (*p*-trend = 0.009) [34]. Participants in the highest quintile of total zinc intake (median: 18.0 mg/day) had a reduced RR of 0.9 (95% CI 0.82, 0.99) for developing Type 2 DM compared to the lowest quintile (median: 4.9 mg/day). In this study, high dietary zinc intake, i.e., excluding supplements, was independently associated with reduction in Type 2 DM risk. Similar findings were reported for a cohort of Australian women where the highest quintile of dietary zinc intake was associated with a 50% decrease in the risk of developing Type 2 DM, compared to the lowest quintile (*p*-trend = 0.006) [32]. 

Conversely, in multivariate analyses, dietary zinc levels were not associated with incidence of Type 2 DM in a cohort of young African American and Caucasian men and women [33]. Similarly, no relationship was observed between dietary zinc intake and the risk of Type 2 DM or metabolic diseases in the Multi-Ethnic Study of Atherosclerosis [30]. In secondary analyses, Otto et al. reported no significant association between zinc intake from red meat and risk of Type 2 DM. The use of zinc supplements was not associated with Type 2 DM [35].

In the Kuopio Ischaemic Heart Disease Risk Factor Study, quartiles of increasing serum zinc levels were positively associated with Type 2 DM incidences (*p*-trend < 0.001) [36]. In the upper quartile of serum zinc concentrations (15.3–24.8 μmol/L), the hazard ratio for developing Type 2 DM was 1.60 (95% CI: 1.20, 2.13) when compared to the reference quartile (serum zinc: 8.3–13.2 μmol/L) after multiple statistical adjustments. The addition of covariates, such as BMI, fasting blood glucose and C-reactive protein, individually did not alter the overall significant trend.

### 3.3. Quality of Included Studies

**Figure 2:**
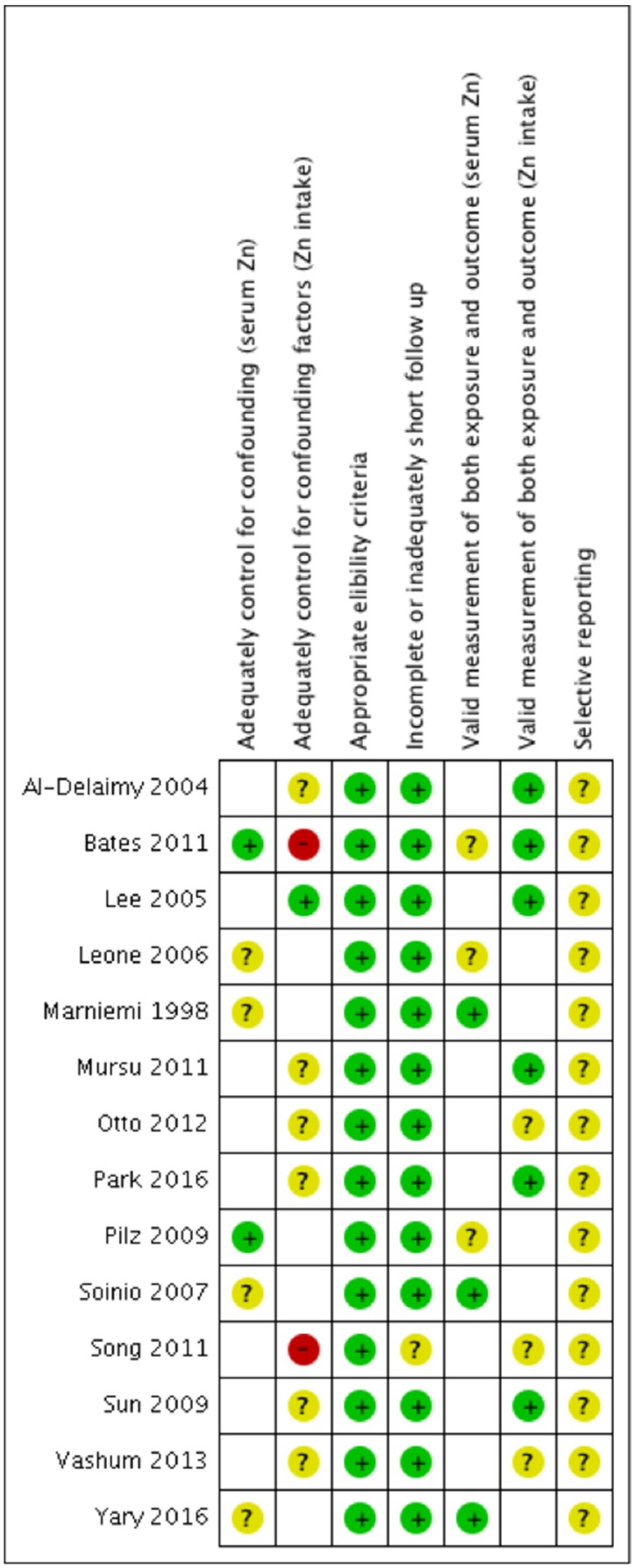
Summary of the risk of biases from each included study. Green (+) symbols represent low risk of bias for the specific criteria within that study. Yellow (?) symbols represent unclear risk of bias and red (−) symbols denote high risk of bias. Support for judgments is presented in Tables S3–S7.

Figure 2 shows the risk of biases of individual studies with support for judgements presented in Tables S3–S7 (Supplementary Materials). All included studies developed and applied appropriate eligibility criteria therefore scoring low risk of bias in the quality assessment. Follow up time and proportion of participant loss to follow up were appropriate for all except one study [35], which had a response rate of 56% at follow up. The included studies scored between low and unclear risk of bias for validity of exposure and outcome measurements. Some of the studies adjusted for confounding factors appropriately, scoring a low or unclear risk of bias. High risk of bias was present in two of the included studies [25,35], where significant confounding factors were not considered. None of the included studies provided access to study protocol or the study protocol did not specify the reported analyses, therefore presenting unclear risk of selective reporting.

## 4. Discussion

The present synthesis of prospective cohort studies has identified an incomplete evidence-base to establish a relationship between zinc intake, CVD and Type 2 DM risk in apparently healthy populations. However, higher serum zinc level appears to be associated with lower risk of CVD in vulnerable populations, such as those with Type 2 DM [23] and patients referred to coronary angiography [31]. 

The prospective cohort studies included in the current review, although limited in number, show no effect of serum zinc levels on CVD risk in nonclinical populations, except in the study of British elderly populations where higher dietary zinc intake was associated with decreased risk of vascular disease mortality following minimal statistical adjustments of age and sex only. In contrast, significant protective effects of zinc status on CVD deaths were shown in those with significant CVD risk factors. Soinio et al. identified that patients with Type 2 DM with serum zinc levels <14.1 μmol/L were at higher risk of MI [23]. Similarly, for patients who were referred to coronary angiography, those with serum zinc <11.9 μmol/L experienced greater risk of CVD mortality [31]. The serum zinc thresholds identified for increased CVD risk from these studies are within the typical reference range of 10–18 μmol/L [37]. This suggests that the optimal level of serum zinc concentrations for the reduction of CVD risk exists at the upper end of the reference range for those with pre-existing risk factors. There is good evidence for improving zinc status in individuals with pre-existing Type 2 DM as an adjunct treatment strategy for the reduction of DM complications, such as CVD [38]. Optimal zinc status, achieved by supplementation or dietary means, is associated with improvements in intermediate markers of Type 2 DM disease progression, such as glycaemic control [10] and lipidemia [11], in addition to reduced rates of complications secondary to DM [23,39]. 

All studies exploring the relationship between zinc intake and prospective risks of CVD and Type 2 DM were conducted in the general population. The evidence of relationship between dietary/supplemental zinc on risk of CVD and Type 2 DM provided by the prospective cohort studies are complicated by inherent confounding factors, such as sex differences. Significant decrease in Type 2 DM incidences were found with higher dietary zinc intake in female participants [32,34], studies involving both sexes with statistical adjustments for sex reported no association [30,35], and in a male study population, higher serum zinc levels were associated with increased risk of Type 2 DM [36]. 

The collation of prospective cohort studies in this review highlights the inconsistency of statistical models utilised in the current literature; this is exemplified by the majority of unclear to high risk of bias ratings for the adequate control of confounding factors. While the majority of the papers included adjustments for confounding factors, the combinations of factors used are largely varied, with some models lacking adjustments that are widely accepted in the literature. For example, while there is good evidence that dietary *trans*-fatty acids are positively associated with CVD risk and events [40], some studies have omitted *trans*-fatty acids as a covariate variable in their models [25,30]. The lack of adjustments for confounding factors can introduce bias in the reported results, especially taken together with the inherent limitations of prospective cohort studies, such as potential selection bias [41,42]. Furthermore, the included studies reported diverse metrics of outcome measures, for example the report of hazard ratios as continuous variable (HR per SD decrease) [25], or comparisons of quartiles [27] and tertiles [28] of serum zinc. The small number of studies, in addition to inconsistencies in statistical models and covariate adjustments, added differences in the determination and presentation of the effects of zinc status on CVD and Type 2 DM rendering meta-analysis of effect inappropriate currently. 

The majority of the studies reporting total zinc intake used FFQ to estimate habitual dietary zinc intake of the study population [24,26,29,30]. In validation studies that compared different methods of dietary assessment, long term zinc intake derived from FFQ and dietary records were well correlated [43]. In contrast, variability in results reported for serum zinc levels may be attributed to differences in methods of measurement. Three of the five included studies [23,27,28] used AAS to determine serum zinc concentration, as the “gold standard” method recommended by the International Zinc Nutrition Consultative Group (IZiNCG) [37]. The other studies [25,31] utilised colorimetric assays for serum zinc analyses, which are liable to significant systematic and fixed bias when compared to zinc analysis by AAS [44]. The intrinsic differences in zinc analysis methods, highlighted in the ratings of risk of biases relating to valid measurements of exposure and outcome, should be considered in the interpretation of variations in the results presented currently. 

The interaction between zinc and other nutrients in determining the risk of cardiometabolic diseases was explored in several included studies [26,32]. Alcohol consumption has been consistently associated with CVD events in a J-shaped relationship, whereby low to moderate alcohol consumption is protective against CVD mortality when compared to non-drinkers or those with high alcohol consumption [45]. When categories of dietary zinc intakes and RR of CVD mortality were stratified by alcohol consumption, Lee et al. showed protective effect of increasing zinc intake for women consuming ≥10 g alcohol/day [26], thereby suggesting additive and separate mechanisms of effect of zinc and alcohol on CVD risk. Interactions between zinc and other minerals, such as iron and copper, have been explored in determining the risk of CVD and Type 2 DM. Higher dietary zinc/iron ratio was associated with lower risk of developing Type 2 DM in a cohort of Australian women [32], possibly due to the antagonist effect of dietary iron on zinc absorption [46]. While known interactions between zinc and other nutrients were explored as ratios or stratified statistical analyses in some included studies, the mechanisms of interaction remain unclear. Furthermore, there is a lack of differentiation in the dietary sources of zinc in the included studies, with only one report of dietary zinc from red meat correlated to CVD risks (but not Type 2 DM risk), despite no association with total zinc intake [30]. Different food groups, such as processed meats, nuts and cereals, have been shown to elicit different effects on risks of CVD and Type 2 DM [47]; therefore, the influence of different food groups on the effects of dietary zinc intake and disease risks should be considered in the evaluation of the relationships. Future investigation should consider the inclusion of secondary analyses determining the effect of dietary sources of zinc on the prospective risks of CVD and Type 2 DM. 

The generalisability of the present results may be limited to high income countries, where all included studies were conducted. Disparities in health care and management of chronic diseases stratified by socioeconomic development of countries are highlighted by differences in life expectancy and probabilities of death from NCD [1]. For individuals with Type 2 DM, management of DM in LMIC may be suboptimal in the prevention of DM associated complications [48]. Novel interventions, such as those that improve zinc status of patients with DM, may be an applicable, low cost strategy, in conjunction with current management therapies, for the prevention of DM complications, such as CVD. In addition, environmental factors that affect zinc levels in soil and water may be important in determining disease risk. Ecological studies have shown negative associations between zinc levels in soil and onset of autoimmune diseases, specifically Type 1 DM [49] and multiple sclerosis [50], suggesting that natural zinc bioavailability in different locations can influence risk of developing diseases. 

Little information regarding the relationship between dietary zinc intake and serum zinc concentration was available from the included studies. A recent meta-regression of zinc supplementation/depletion studies revealed a logarithmic relationship between these two variables, whereby a doubling of zinc intake corresponded with a 6% increase in serum zinc concentration [51]. However, this relationship is complicated by the numerous factors, other than zinc intake, that can influence serum zinc concentration [46], for example, inflammation and zinc bioavailability. Reduced zinc bioavailability, in particular in diets with a high ratio of phytate to zinc, has been suggested to impair the absorption of zinc in the gastrointestinal tract and hence incorporation into body tissues [52]. However, it is difficult to determine the absolute amount of zinc absorbed as it is likely that the higher zinc content in some high phytate foods, compared to products lower in phytate, may compensate for the less efficient absorption of zinc [53].

Furthermore, potential interactions should be considered in regards to the effects of current preventative and treatment recommendations for obesity-related chronic diseases, such as exercise and dietary management, on zinc status. For example, higher intakes of dietary fibre are often recommended to individuals with Type 2 DM [54] and those with increased risk for cardiometabolic diseases. Foods that are high in fibre are also high in phytate and the effects of phytate on reducing the bioavailability of zinc in the gut is well-established [55]; thereby, the incidental effect of increased dietary fibre on risk of zinc deficiency may be a determining factor in risk of chronic diseases. Moreover, lifestyle modifications, specifically physical activity, can modulate nutrient status [56] with implications for beneficial effects induced by exercise [57]. Further investigations are required to examine the interactions between zinc status and current preventative and treatment recommendations, especially exercise and intakes of competing nutrients, in the management of obesity-related chronic diseases. 

## 5. Conclusions

To the best of our knowledge, the current report is the first systematic review of prospective cohort studies assessing the relationship between zinc status and risks of CVD and Type 2 DM. The strengths of the present study lie in the systematic collation of available evidence and the determination of risk of study biases in line with current GRADE approach adopted by the Cochrane Collaboration [21]. While the available data did not allow for meta-analysis of effect, the qualitative assessment of the current evidence suggests a protective effect of zinc in the development of cardiometabolic diseases, specifically CVD. The effect of zinc appears to be more pronounced in vulnerable populations, such as those with existing Type 2 DM or established risk factors of diseases. Further investigations into the mechanisms of zinc’s action on the pathogenesis of chronic diseases and additional evidence from observational studies, in particular the influence of dietary zinc bioavailability, differentiation in sources of zinc and appropriate statistical adjustments, are required for the establishment of dietary zinc recommendations in the prevention of CVD and Type 2 DM.

## Figures and Tables

**Table 1:** Relationship between dietary and/or supplemental zinc intake and cardiovascular disease events in prospective cohort studies.

Authors, Country	Population	Baseline Age (Year) ^1^	Sex	Follow up (Year)	Total *n* (No. of Cases)	Adjustments	Disease Outcome	Outcome Summary
Al-Delaimy et al. 2004 [24], USA	Health professionals, excluded participants with cancer, MI and CVD	40–75	Male	12	39,633 (1449)	Age, energy	Trend (*p* = 0.06) towards increased risk of CHD with higher total Zn intake (dietary and supplemental), however no effect in the 5th quintile (median dietary Zn = 37 mg/day; RR 0.96, 95% CI 0.81, 1.14).	No association
						Age, time period, energy intake, history of diabetes, history of high cholesterol, family history of MI, smoking history, aspirin intake, BMI, alcohol intake, physical activity, dietary *trans* fatty acid, Vitamin E, total protein intake, cereal fiber, folate, omega 3 fatty acids	No association between total Zn intake and risk of CHD (RR in the 5th quintile: 1.07; 95% CI 0.87, 1.3; *p*-trend = 0.93) or when separated into fatal CHD or non-fatal CHD.	No association
						Age, energy	No association between Zn supplement dose and risk of CHD (RR in the 5th quintile: 0.91; 95% CI: 0.69, 1.2) or when separated into fatal CHD or non-fatal CHD.	No association
						Age, time period, energy intake, history of diabetes, history of high cholesterol, family history of MI, smoking history, aspirin intake, BMI, alcohol intake, physical activity, dietary *trans* fatty acid, Vitamin E, total protein intake, cereal fiber, folate, omega 3 fatty acids	No association between Zn supplement dose and risk of CHD (RR in the 5th quintile: 1.06; 95% CI 0.79, 1.43) or when separated into fatal CHD or non-fatal CHD.	No association
Bates et al. 2011 [25], UK	Community-living age ≥ 65 year	76.6 ± 7.4	49% Female	14	1054 (189)	Age, sex	Higher dietary Zn intake was associated with decreased risk of vascular disease mortality ^3^ (HR 0.84 per SD increase, 95% CI 0.71, 0.99; *p* = 0.04). Mean Zn intakes were 8.81 ± 2.86 mg/day for males and 6.96 ± 2.56 mg/day for females (mean ± SD).	Reduced risk
Lee et al. 2005 [26] ^2^, USA	Postmenopausal women and no report of angina, heart disease or heart attack at baseline	55–69	Female	15	34,492 (1767)	Age, total energy intake, history of hypertension, BMI, waist-hip ratio, physical activity, cigarette smoking, alcohol consumption, hormone replacement therapy, intakes of saturated fat, *trans* fat, PUFA, folate, β-carotene, Vitamin C and Vitamin E	No association between dietary Zn intake and risk of CVD mortality, when stratified into alcohol consumption or in combined analysis. In participants with alcohol consumption of 10–29 g/day, trend for a reduction in CVD risk with higher dietary Zn intake in participants (5th quintile RR 0.37, 95% CI 0.13,1.06; *p*-trend = 0.07).	No association
Mursu et al. 2011 [29] ^2^, USA	Mostly postmenopausal women	61.6 ± 4.2	Female	22 (mean 19)	37,033 (5454)	Age, energy intake	No association between Zn supplement use and CVD mortality (HR for users 0.97; 95% CI 0.91, 1.03).	No association
					37,033 (5454)	Age, energy intake, education, place of residence, diabetes, hypertension, BMI, waist-hip ratio, hormone replacement therapy, physical activity, smoking, intake of alcohol, saturated fat, whole grain products, fruits and vegetables	No association between Zn supplement use and CVD mortality (HR for users 1.08; 95% CI 1.01, 1.15).	No association
Otto et al. 2012 [30], USA	Population-based sample, free of clinical CVD	61.8 ± 10.3 (SE)	53% Female	6.2	5285 (8.5 new cases per 1000 person-years)	Energy intake, age, sex, race-ethnicity, education, study center, alcohol intake, physical activity, BMI, fiber intake, cigarette smoking, dietary supplement use	No association between dietary Zn intake and risk of CVD (*p*-trend = 0.46, 5th quintile Zn intake ≥ 9.8 mg/day). Higher Zn intake from red meat was associated with increased risk of CVD (5th quintile HR 1.51, 95% CI 1.02, 2.24; *p*-trend = 0.01). No association between Zn intake from other sources and risk of CVD.	No association
					5285 (8.5 new cases per 1000 person-years)	Energy intake, age, sex, race-ethnicity, education, study center, alcohol intake, physical activity, BMI, fiber intake, cigarette smoking, dietary supplement use, PUFA:SFA, intake of Mg, nonheme iron, heme iron, β-carotene, Vitamin E and Vitamin C	No association between dietary Zn intake and risk of CVD (*p*-trend = 0.66). Higher Zn intake from red meat was associated with increased risk of CVD (5th quintile HR 1.66, 95% CI 1.10, 2.49; *p*-trend < 0.01). No association between Zn intake from other sources and risk of CVD.	No association

^1^ Age given as mean ± SD or range (unless otherwise specified); ^2^ Reports derived from Iowa’s Women’s Health Study; ^3^ as defined by International Classification of Disease, includes deaths from stroke. CHD, coronary heart disease; CI, confidence interval; CVD, cardiovascular disease; HR, hazard ratio; MI, myocardial infarction; PUFA, polyunsaturated fatty acids; RR, relative risk; SFA, saturated fatty acids.

**Table 2:** Relationship between serum zinc levels and cardiovascular disease events in prospective cohort studies.

Authors, Country	Population	Baseline Age (Year) ^1^	Sex	Follow up (Year)	Total *n* (No. of Cases)	Adjustments	Disease Outcome	Outcome Summary
Bates et al. 2011 [25], UK	Community-living age ≥ 65 year	76.6 ± 7.4	49% Female	14	741 (189)	Age, sex	Higher plasma Zn concentration was associated with reduced risk of vascular disease mortality ^2^ (HR 0.73 per SD increase, 95% CI 0.61, 0.88; *p* = 0.001). Mean plasma Zn levels were 14.2 ± 2.1 μmol/L for males and 14.2 ± 2.4 μmol/L for females (mean ± SD).	Reduced risk
					629 (105)	Age, sex, vitamin and mineral predictors, α_1_-antichymotrypsin, creatinine, total and HDL-cholesterol, albumin, BMI, SBP, smoking, No. of prescribed drugs, self-reported health, physical activity, poverty	No association between plasma Zn concentration and risk of vascular disease mortality (HR 0.83 per SD increase, 95% CI 0.65, 1.07; *p* = 0.15).	No association
Leone et al. 2006 [27], France	Men aged ≥ 30 year at CVD screening	30–60	Male	18	4035 (56)	Age, BMI, smoking, alcohol consumption, physical activity, hypertension, serum LDL, HDL and triglycerides, diabetes, and CVD history	No association between serum Zn level and CVD death (RR in the 4th quartile 0.7; 95% CI 0.3, 1.5). Mean serum Zn levels were 14.6 ± 1.8 μmol/L for survivors and 14.5 ± 2.1 μmol/L for deceased.	No association
Marniemi et al. 1998 [28], Finland	Community-living age ≥ 65 year	≥65	47% Female	13	344 (142)	Age, sex, smoking, alcohol use, BMI, coronary heart diseases, hypertension, diabetes, serum total and HDL-cholesterol, TAG	No association between serum Zn level and vascular mortality (RR for 3rd tertile 1.17; 95% CI 0.74, 1.84). Mean serum Zn levels were 12.9 ± 1.7 μmol/L for survivors and 12.9 ± 1.9 μmol/L for deceased by vascular death.	No association
Pilz et al. 2009 [31], Germany	Clinically stable patients of German ancestry who were referred to coronary angiography	>60 (median)	30% Female	7.75 (median)	3274 (484)	Unadjusted	Lower serum Zn level was associated with increased CVD mortality (per quartile decrease HR 1.30, 95% CI 1.19, 1.41; *p* < 0.001). Higher risk of CVD mortality for 1st quartile (serum Zn < 11.93 μmol/L; HR 2.12, 95% CI 1.63, 2.77; *p* < 0.001).	Reduced risk
					3274 (484)	Age, sex, BMI, HbA1c, systemic hypertension, smoking, HDL and LDL-cholesterol, TAG, GFR, CRP, N-terminal pro-B-type natriuretic peptide, copper, albumin, Hb, homocysteine, ACE inhibitors, diuretics	Lower serum Zn level was associated with increased CVD mortality (per quartile decrease HR 1.10, 95% CI 1.01, 1.21; *p* = 0.038). No association in CVD mortality for 1st quartile (serum Zn < 11.93 μmol/L; HR 1.24, 95% CI 0.92, 1.66; *p* = 0.162).	Reduced risk
Soinio et al. 2007 [23], Finland	Patients with Type 2 DM	45–64	45% Female	7	1050 (156 CHD deaths)	Unadjusted	Higher baseline serum Zn level was associated with reduction in risk of CHD death (*p* = 0.015). Participants in the lowest quartile (≤14.1 μmol/L) have increased risk of CHD death than those in the upper 3 quartiles (RR 1.80, 95% CI 1.30, 2.49, *p* < 0.001).	Reduced risk
					1050 (156 CHD deaths)	Age, sex, duration of diabetes, cholesterol (total and HDL), TAG, HbA1c, eGFR, hypertension, smoking, BMI, area of residence, type of diabetes therapy	Participants in the lowest quartile of serum Zn level (≤14.1 μmol/L) have increased risk of CHD death than those in the upper 3 quartiles (RR 1.70, 95% CI 1.21, 2.38, *p* = 0.002) ^2^.	Reduced risk
					1050 (254 fatal or non-fatal MI)	Unadjusted	Higher baseline serum Zn level was associated with reduction in risk of MI (*p* = 0.014). Participants in the lowest quartile (≤14.1 μmol/L) have increased risk of CHD death or nonfatal MI than those in the upper 3 quartiles (RR 1.40, 95% CI 1.06, 1.84, *p* = 0.019).	Reduced risk
					1050 (254 fatal or non-fatal MI)	Age, sex, duration of diabetes, cholesterol (total and HDL), TAG, HbA1c, eGFR, hypertension, smoking, BMI, area of residence, type of diabetes therapy	Participants in the lowest quartile of serum Zn level (≤14.1 μmol/L) have increased risk of MI than those in the upper 3 quartiles (RR 1.37, 95% CI 1.03, 1.82, *p* = 0.033) ^2^.	Reduced risk

^1^ Age given as mean ± SD or range (unless otherwise specified); ^2^ Models remain significant after addition of CRP as a covariate (authors did not provide sufficient data for extraction). ACE, angiotensin converting enzyme; CHD, coronary heart disease; CI, confidence interval; CRP, C-reactive protein; CVD, cardiovascular disease; GFR, glomerular filtration rate; HDL, high density lipoprotein; HR, hazard ratio; RR, relative risk; SBP, systolic blood pressure; TAG, triglycerides.

**Table 3:** Relationship between zinc status and risk of Type 2 diabetes mellitus in prospective cohort studies.

Authors, Country	Population	Baseline Age (Year) ^1^	Sex	Follow up (Year)	Total *n* (No. of Cases)	Marker of Zn Status	Adjustments	Disease Outcome	Outcome Summary
Otto et al. 2012 [30], USA	Population-based sample, free of clinical CVD and Type 2 DM at baseline	45–84	53%	4.8	4982 (16.7 new cases per 1000 person-years)	Dietary Zn	Energy intake, age, sex, race-ethnicity, education, study center, alcohol intake, physical activity, BMI, fiber intake, cigarette smoking, dietary supplement use	No association between dietary Zn intake and risk of Type 2 DM (5th quintile HR 1.15; 95% CI 0.8, 1.63; *p* = 0.71). No association between Zn intake from red meat and risk of Type 2 DM.	No association
							Energy intake, age, sex, race-ethnicity, education, study center, alcohol intake, physical activity, BMI, fiber intake, cigarette smoking, dietary supplement use, PUFA:SFA, intake of Mg, nonheme iron, heme iron, β-carotene, vitamin E and vitamin C	No association between dietary Zn intake and risk of Type 2 DM (5th quintile HR 1.41; 95% CI 0.88, 2.27; *p* = 0.33). No association between Zn intake from red meat and risk of Type 2 DM.	No association
Park et al. 2016 [33], USA	African American and Caucasian men and women	27.03 ± 3.61	52.5% Female	23	3960 (418)	Total Zn intake	Age, gender, ethnicity, study center, BMI, baseline HOMA-IR	No association between total zinc intake (dietary + supplement) and risk of Type 2 DM (4th quartile HR 0.98; 95% CI 0.75, 1.27; *p* = 0.97).	No association
							Age, gender, ethnicity, study center, BMI, baseline HOMA-IR, education, smoking, alcohol consumption, physical activity, family history of diabetes, intakes of long-chain omega 3 PUFA, Mg, iron and total energy	No association between total zinc intake (dietary + supplemental) and risk of Type 2 DM (4th quartile HR 1.27; 95% CI 0.81, 2.01; *p* = 0.23).	No association
Song et al. 2011 [35], USA	AARP ^2^ members free of diabetes in the initial 4–5 years of follow up (2000)	50–71	42% Female	8–11	232,007 (14,130)	Zn supplement use	Age, sex, race, BMI, education, marital status, physical activity, smoking, coffee consumption, alcohol, general health, total energy intake, multivitamin use, individual vitamin and minerals use and frequency	No association between Zn supplement use with risk of Type 2 DM (users OR 0.94; 95% CI 0.86, 1.03; *p* = 0.16).	No association
Sun et al. 2009 [34], USA	Nurses free of diabetes, cancer or CVD at baseline	33–60	Female	24	82,297 (6030)	Dietary Zn	Age	Higher total Zn intake (dietary + supplement) was associated with reduced risk of Type 2 DM (5th quintile RR 0.83; 95% CI 0.77, 0.9; *p*-trend < 0.0001). No association between dietary Zn intake and risk of Type 2 DM (5th quintile RR 1.00; 95% CI 0.92, 1.08; *p*-trend = 0.04).	Reduced risk
							Age, BMI, family history of diabetes, smoking, alcohol intake, menopausal status, postmenopausal hormone use, multivitamin use, physical activity, total energy intake, glycaemic load, PUFA:SFA, intakes of red meat, heme iron, whole grains, trans fat, Mg and caffeine (Zn intake from supplement use in tertiles was further adjusted when modeling the associations for dietary Zn intake)	Higher total Zn intake (dietary + supplement) was associated with reduced risk of Type 2 DM (5th quintile RR 0.9; 95% CI 0.82, 0.99; *p*-trend = 0.04). Higher dietary Zn intake was associated with reduced risk of Type 2 DM (5th quintile RR 0.92; 95% CI 0.84, 1.00; *p*-trend = 0.009).	Reduced risk
Vashum et al. 2013 [32], Australia	Women	45–50	Female	6	8921 (333)	Dietary Zn	Energy, age	No association between dietary Zn intake and risk of Type 2 DM (5th quintile OR 0.75; 95% CI 0.53, 1.05; *p*-trend = 0.319).	No association
							Energy, age, BMI, smoking, hormone replacement therapy, exercise, medical history of arthritis, CHD, hypertension, asthma and depression, energy adjusted fiber, iron and fat intake, alcohol and supplement use	Higher levels of dietary Zn intake was associated with reduced risk of Type 2 DM (5th quintile OR 0.50, 95% CI 0.32, 0.77, *p*-trend = 0.006).	Reduced risk
Yary et al. 2016 [36], Finland	Finnish men	42–60	Male	20	2220 (416)	Serum Zn	Age, examination year	Positive association between serum Zn quartiles and incidence of Type 2 DM (4th quartile HR 1.52; 95% CI 1.15, 2.01; *p*-trend = 0.001).	Increased risk
							Age, examination year, family history of DM, smoking, education years, leisure-time physical activity, intake of alcohol, fiber, sum of fruits, berries and vegetables	Positive association between serum zinc quartiles and incidence of Type 2 DM (4th quartile HR 1.60; 95% CI 1.20, 2.13; *p*-trend < 0.001). Adjustments for BMI, fasting blood glucose, serum insulin, HOMA-IR, HOMA-IS, HOMA-β or CRP individually, did not affect the statistical significant *p*-trend.	Increased risk

^1^ Age given as mean ± SD or range (unless otherwise specified); ^2^ AARP, American Association of Retired Persons; CHD, coronary heart disease; CI, confidence interval; CRP, C-reactive protein; CVD, cardiovascular disease; DM, diabetes mellitus; HOMA-IR, homeostatic model assessment–insulin resistance; OR, odds ratio; HOMA-IS, homeostatic model assessment–insulin sensitivity; HOMA-β, homeostatic model assessment–β-function; HR, hazard ratio; PUFA, polyunsaturated fatty acids; RR, relative risk; SFA, saturated fatty acids.

**Table 4:** Summary of relationships between increasing zinc status and prospective risks of cardiovascular diseases and Type 2 diabetes mellitus.

	Study ID	Prospective Risk of CVD	Prospective Risk of Type 2 DM
Zinc intake	Al-Delaimy et al. 2004 [24]	No association	N/A
	Bates et al. 2011 [25]	↓ risk	N/A
	Lee et al. 2005 [26]	No association	N/A
	Leone et al. 2006 [27]	No association	N/A
	Park et al. 2016 [33]	N/A	No association
	Otto et al. 2012 [30]	No association	No association
	Sun et al. 2009 [34]	N/A	↓ risk
	Vashum et al. 2013 [32]	N/A	↓ risk
Zinc supplement	Al-Delaimy et al. 2004 [24]	No association	N/A
	Mursu et al. 2011 [29]	No association	N/A
	Song et al. 2011 [35]	N/A	No association
Plasma/serum zinc	Bates et al. 2011 [25]	↓ risk/no association	N/A
	Leone et al. 2006 [27]	No association	N/A
	Marniemi et al. 1998 [28]	No association	N/A
	Pilz et al. 2009 [31]	↓ risk	N/A
	Soinio et al. 2007 [23]	↓ risk	N/A
	Yary et al. 2016 [36]	N/A	↑ risk

↑, Increased; ↓, decreased.

## Supplementary Materials

The following are available online at http://www.mdpi.com/2072-6643/8/11/707/s1, Table S1. Full search strategy used in EMBASE for the search terms (zinc AND [(dietary OR supplement*) OR (plasma OR serum)] AND diabetes), Table S2. Risk of bias assessment of included studies, Table S3. Scoring criteria of confounding factors and results of each individual study with models describing zinc intake and CVD outcomes, Table S4. Scoring criteria of confounding factors and results of each individual study with models describing serum zinc concentration and CVD outcomes, Table S5. Scoring criteria of confounding factors and results of each individual study with models describing zinc intake and Type 2 DM outcomes, Table S6. Scoring criteria of confounding factors and results of each individual study with models describing zinc intake and Type 2 DM outcomes, Table S7. Scoring criteria of confounding factors and results of each individual study with models describing serum zinc concentration and Type 2 DM outcomes.
