# Supplementary material for: Zinc Status and Risk of Cardiovascular Diseases and Type 2 Diabetes Mellitus—A Systematic Review of Prospective Cohort Studies
Source: Nutrients. 2016 Nov 5;8(11):707. doi: 10.3390/nu8110707 (PMC5133094; doi:10.3390/nu8110707)
Supplement: Supplementary file 1 [file nutrients-08-00707-s001.docx]

Supplementary Materials: Zinc Status and Risk of Cardiovascular Diseases and Type 2 Diabetes Mellitus—A Systematic Review of Prospective Cohort Studies

Anna Chu, Meika Foster and Samir Samman

**Table S1.** Full search strategy used in EMBASE for the search terms (zinc AND [(dietary OR supplement*) OR (plasma OR serum)] AND diabetes).

| 1 | zinc.mp. or exp zinc/ | 182,532 |
| --- | --- | --- |
| 2 | dietary.mp. or exp dietary intake/ | 546,655 |
| 3 | exp diet supplementation/ or supplement*.mp. or exp diet/ | 567,582 |
| 4 | plasma/ or plasma.mp. | 1,031,803 |
| 5 | serum/ or serum.mp. | 1,200,860 |
| 6 | 2 or 3 or 4 or 5 | 2,846,464 |
| 7 | diabetes.mp. or exp diabetes mellitus/ | 785,297 |
| 8 | 1 and 6 and 7 | 2682 |
| 9 | limit 8 to (human and english language) | 1601 |

**Table S2.** Confounding factors and associated scores for risk of bias assessment.

| **Model Variables  (Maximum Total Score)** | **Confounding Factor** | **Maximum Individual Score** |
| --- | --- | --- |
| Zinc intake and CVD (5) | Family history | 0.5 |
|  | Co-morbidities | 0.5 |
|  | Lifestyle factors | 0.5 |
|  | Anthropometry | 0.5 |
|  | Dietary *trans* fatty acid | 1 |
|  | Dietary saturated fat | 1 |
|  | Other dietary factors | 0.5 |
|  | Medication | 0.5 |
| Serum zinc and CVD (3) | Total blood cholesterol or LDL-cholesterol | 1 |
|  | HDL-cholesterol | 1 |
|  | Inflammatory biomarker | 1 |
| Zinc intake and Type 2 DM (5) | Family history | 0.5 |
|  | Co-morbidities | 0.5 |
|  | Lifestyle factors | 0.5 |
|  | Anthropometry | 0.5 |
|  | Dietary fibre intake | 1 |
|  | Saturated fat intake | 1 |
|  | Other dietary factors | 0.5 |
|  | Medication | 0.5 |
| Serum zinc and Type 2 DM (3) | Fasting glucose or HbA1c | 1 |
|  | HDL-cholesterol | 0.5 |
|  | Triglycerides | 0.5 |
|  | Inflammatory biomarker | 1 |

**Table S3.** Risk of bias assessment of included studies

| **Al-Delaimy et al. 2004** **[1]** | | |
| --- | --- | --- |
| **Item** | **Authors’ Judgement** | **Support for Judgement** |
| Adequately control for  confounding factors (serum Zn) | N/A | N/A |
| Adequately control for  confounding factors (Zn intake) | Unclear risk | Score of 4/5 for confounding  criteria. Multi-nutrient  models did not adjust for  saturated fat intake. |
| Appropriate eligibility criteria | Low risk | Initial sample population  from a wide range of health  professions in the US,  represented by all states  with no exclusion for race.  Authors excluded participants  who failed to report  plausible dietary data. |
| Incomplete or inadequately short follow up | Low risk | The follow-up was maintained  for 12 years with an average of  94% follow-up rate. |
| Valid measurements of  both exposure and outcome (serum Zn) | N/A | N/A |
| Valid measurements of  both exposure and outcome (Zn intake) | Low risk | The study used a 131-item  semi-quantitative food  frequency questionnaire.  Deaths were reported by  next-of-kin, co-workers,  postal authorities or in the  National Death Index. |
| Selective reporting | Unclear risk | No study protocol identified. |
| **Bates et al. 2011 [2]** | | |
| Adequately control for  confounding factors (serum Zn) | Low risk | Score of 3/3 for confounding  criteria. Appropriate  adjustment of confounding factors. |
| Adequately control for  confounding factors (Zn intake) | High risk | In the model of dietary zinc  intake and vascular disease  mortality, the authors only  adjusted for age and sex. |
| Appropriate eligibility criteria | Low risk | Participants were drawn  from eight randomly  selected postcode sectors in  mainland Britain. |
| Incomplete or inadequately short follow up | Low risk | Follow up maintained for  14 years with 94.5%  follow up rate. |
| Valid measurements of  both exposure and outcome (serum Zn) | Unclear risk | Colorimetric assay used for  plasma zinc concentration,  which is not the gold standard  zinc measurement method. |
| Valid measurements of  both exposure and outcome (Zn intake) | Low risk | 4-day weighed dietary record for  zinc intake. National Register  of Births and Deaths was used  to document cause of death. |
| Selective reporting | Unclear risk | No study protocol identified |
| **Lee et al. 2005 [3]** | | |
| Adequately control for  confounding factors (serum Zn) | N/A | N/A |
| Adequately control for  confounding factors (Zn intake) | Low risk | Score of 4.5/5 for confounding  factors. Appropriate  adjustments of  confounding factors. |
| Appropriate eligibility criteria | Low risk | 41,836 women, aged 55–69 years,  selected randomly from Iowa  Department of Transportation  driver’s license list. |
| Incomplete or inadequately short follow up | Low risk | Follow up of 15 years,  investigators excluded 18%  of initial sampled  population with reasons |
| Valid measurements of  both exposure and outcome (serum Zn) | N/A | N/A |
| Valid measurements of  both exposure and outcome (Zn intake) | Low risk | Deaths for subjects were  found through the National  Death Index. 127 items food  frequency questionnaire was  used to determine zinc intake |
| Selective reporting | Unclear risk | Models were stratified by  levels of alcohol consumption |
| **Leone et al. 2006 [4]** | | |
| Adequately control for  confounding factors (serum Zn) | Unclear risk | Score of 2/3 for confounding  factors. Models did not adjust  for CRP or other acute phase  inflammatory indicators. |
| Adequately control  for confounding  factors (Zn intake) | N/A | N/A |
| Appropriate eligibility criteria | Low risk | The study population  involved male employees of  3 large public organisation  in Paris who volunteered for  a cardiovascular screening. |
| Incomplete or inadequately short follow up | Low risk | Follow up of 18 years, < 2% of  participants lost to follow up. |
| Valid measurements of  both exposure and outcome (serum Zn) | Unclear risk | Mortality follow up  determined by death  certificate and cause of death  registered by the National  Institute of Health and  Medical Research. Zinc was  determined by flame AAS  with fasting blood samples. |
| Valid measurements of  both exposure and outcome (Zn intake) | N/A | N/A |
| Selective reporting | Unclear risk | No study protocol identified. |
| **Marniemi et al. 1998 [5]** | | |
| Adequately control for  confounding factors (serum Zn) | Unclear risk | Score of 2/3 for confounding  factors. Models did not adjust  for inflammatory markers. |
| Adequately control for  confounding factors (Zn intake) | N/A | N/A |
| Appropriate eligibility criteria | Low risk | Random sample of 480  community-living elderly individuals |
| Incomplete or inadequately short follow up | Low risk | Follow up of 13 years,  all participants appear to  be accounted for. |
| Valid measurements of  both exposure and outcome (serum Zn) | Low risk | Mortality were recorded  from the National Death  Register. Serum zinc were  analysed by direct flame  AAS from morning fasting  blood samples. |
| Valid measurements of  both exposure and outcome (Zn intake) | N/A | N/A |
| Selective reporting | Unclear risk | No study protocol identified. |
| **Mursu et al. 2011 [6]** | | |
| Adequately control for  confounding factors (serum Zn) | N/A | N/A |
| Adequately control for  confounding factors (Zn intake) | Unclear risk | Score of 3.5/5 for confounding  factors. Models did not  adjust for trans fat intake. |
| Appropriate eligibility criteria | Low risk | 41,836 women, aged 55–69 years,  selected randomly from  Iowa Department of  Transportation driver’s license list. |
| Incomplete or inadequately short follow up | Low risk | Mean follow up of 19 years,  29% of cohort at baseline  excluded due to loss to  follow up and implausible  dietary intake. |
| Valid measurements of  both exposure and outcome (serum Zn) | N/A | N/A |
| Valid measurements of  both exposure and outcome (Zn intake) | Low risk | Deaths identified through  State or National Death  Registry. Supplement use was  queried in 3 surveys during 19  years. Validated 127-food item  FFQ was used to  determine zinc intake. |
| Selective reporting | Unclear | No study protocol identified. |
| **Otto et al. 2012 [7]** | | |
| Adequately control for  confounding factors (serum Zn) | N/A | N/A |
| Adequately control for  confounding factors (Zn intake) | Unclear risk | Score of 2.5/5 for CVD;  authors did not include trans  fat intake as confounding factor.  Score of 2.5/5 for Type 2 DM;  authors did not include  physical activity or  saturated fat intake as  confounding factors. |
| Appropriate eligibility criteria | Low risk | Total of 6814 adults recruited  from six US communities. |
| Incomplete or inadequately short follow up | Low risk | Mean follow up of 6.2 years for  CVD and 4.8 years for type 2 DM |
| Valid measurements of  both exposure and outcome (serum Zn) | N/A | N/A |
| Valid measurements of  both exposure and outcome (Zn intake) | Unclear risk | Assessment of incident  diabetes by self-reported  diagnosis, serum glucose > 7  mmol/L or new use of  hypoglycaemic medication.  Assessment of CVD  incidences was defined by  self-reported, from medical  record or obituaries reviewed  by a medical end point committee.  127-item FFQ was  used for zinc intake. |
| Selective reporting | Unclear risk | Study protocol identified.  Protocol did not include  analysis described in the paper. |
| **Park et al. 2016 [8]** | | |
| Adequately control for  confounding factors (serum Zn) | N/A | N/A |
| Adequately control for  confounding factors (Zn intake) | Unclear risk | Score of 2/5 for Type 2 DM;  authors did not include  saturated fat intake or dietary  fibre as confounding factors. |
| Appropriate eligibility criteria | Low risk | Total of 5114 adults  recruited from 4 US cities |
| Incomplete or inadequately short follow up | Low risk | 68.4% follow up rate at  23 years follow up. |
| Valid measurements of  both exposure and outcome (serum Zn) | N/A | N/A |
| Valid measurements of  both exposure and outcome (Zn intake) | Low risk | Dietary zinc assessed by  validated diet history  questionnaire; Type 2 DM  determined by objective  outcomes (plasma glucose,  OGTT or HbA1c) |
| Selective reporting | Unclear risk | Study protocol identified.  Protocol did not  include analysis  described in the paper. |
| **Pilz et al. 2009 [9]** | | |
| Adequately control for  confounding factors (serum Zn) | Low risk | Score of 3/3 for confounding  factors; adequately controlled  for confounding factors. |
| Adequately control for  confounding factors (Zn intake) | N/A | N/A |
| Appropriate eligibility criteria | Low risk | 3316 patients referred for  coronary angiography  at one hospital in  Southwest Germany. |
| Incomplete or inadequately short follow up | Low risk | Median follow up of 7.75 years,  0.5% patients lost to follow up. |
| Valid measurements of  both exposure and outcome (serum Zn) | Unclear risk | Medical records, death  certificates and autopsy data  to determine causes of death.  Serum zinc was determined  by colorimetric method using  fasting blood samples. |
| Valid measurements of  both exposure and outcome (Zn intake) | N/A | N/A |
| Selective reporting | Unclear risk | Study protocol was identified.  Statistical plan described  to be exploratory. |
| **Soinio et al. 2007 [10]** | | |
| Adequately control for  confounding factors (serum Zn) | Unclear risk | Score of 2/3 for confounding  factors; authors did not adjust  for CPR in models presented  in Table 2. Authors described  in text that models with CRP  did not change the  results from Table 2. |
| Adequately control for  confounding factors (Zn intake) | N/A | N/A |
| Appropriate eligibility criteria | Low risk | Patients with type 2 DM,  living in two districts in  Finland, identified through  a national drug  reimbursement register. |
| Incomplete or inadequately short follow up | Low risk | Follow up of 7 years;  no loss to follow up |
| Valid measurements of  both exposure and outcome (serum Zn) | Low risk | Questionnaire to all surviving  patients asking about  hospitalisation for acute chest  pain and review of medical  records of participants,  who died or reported  hospitalisation.  Serum zinc levels were  determined by AAS 10 years  later from stored samples. |
| Valid measurements of  both exposure and outcome (Zn intake) | N/A | N/A |
| Selective reporting | Unclear risk | No study protocol identified. |
| **Song et al. 2011 [11]** | | |
| Adequately control for  confounding factors (serum Zn) | N/A | N/A |
| Adequately control for  confounding factors (Zn intake) | High risk | Score of 1.5/5 for confounding  factors; authors did not  adjust for any aspects of  family history, comorbidities  and saturated fat or  dietary fibre intakes. |
| Appropriate eligibility criteria | Low risk | Cohort participants  (*n* = 566,402) were recruited  by mail out of all AARP  members from six states and  two metropolitan areas. |
| Incomplete or inadequately short follow up | Unclear risk | Follow up of 8–11 years; 56%  response rate on follow up. |
| Valid measurements of  both exposure and outcome (serum Zn) | N/A | N/A |
| Valid measurements of  both exposure and outcome (Zn intake) | Unclear risk | Self reported vitamin  and mineral use and  diabetes diagnosis in  follow up questionnaires. |
| Selective reporting | Unclear risk | Study protocol identified;  no specific mention of zinc  or zinc supplement. |
| **Sun et al. 2009 [12]** | | |
| Adequately control for  confounding factors (serum Zn) | N/A | N/A |
| Adequately control for  confounding factors (Zn intake) | Unclear risk | Score of 2.5/5 for confounding  factors; authors did not  adjust for saturated fat  or dietary fibre intakes. |
| Appropriate eligibility criteria | Low risk | Names and address of  registered nurses in the  American Nurse Association  were obtained and invited  to participate. |
| Incomplete or inadequately short follow up | Low risk | Follow up of 24 years;  95% follow up rate. |
| Valid measurements of both  exposure and outcome (serum Zn) | N/A | N/A |
| Valid measurements of both  exposure and outcome (Zn intake) | Low risk | Self report of diabetes  diagnosis according to  the criteria of National  Diabetes Data Group and  American Diabetes  Association (Participants  are/were Registered Nurses).  FFQ was used to determine  dietary intakes. |
| Selective reporting | Unclear risk | No study protocol identified. |
| **Vashum et al. 2013 [13]** | | |
| Adequately control for  confounding factors (serum Zn) | N/A | N/A |
| Adequately control for  confounding factors (Zn intake) | Unclear risk | Score of 3.5/5 for confounding  factors; authors did not adjust  for saturated fat intake or  family history. |
| Appropriate eligibility criteria | Low risk | Women selected randomly  within each age group from  the National Medicare Health  Insurance Database with  intentional overrepresentation  of women living in rural and  remote areas. |
| Incomplete or inadequately short follow up | Low risk | Follow up of 6 years with a  response rate of 83% |
| Valid measurements of both  exposure and outcome (serum Zn) | N/A | N/A |
| Valid measurements of both  exposure and outcome (Zn intake) | Unclear risk | Self report of diabetes  diagnosis. FFQ was used to  determine dietary intakes. |
| Selective reporting | Unclear risk | No study protocol identified. |
| **Yary et al. 2016 [14]** | | |
| Adequately control for  confounding factors (serum Zn) | Unclear risk | Score of 2/3 for confounding  factors; authors did not adjust  for HDL-cholesterol or  triglycerides. |
| Adequately control for  confounding factors (Zn intake) | N/A | N/A |
| Appropriate eligibility criteria | Low risk | Population-based randomly  selected sample of men from  Eastern Finland |
| Incomplete or inadequately short follow up | Low risk | 80% follow up rate at  20 years follow up |
| Valid measurements of both  exposure and outcome (serum Zn) | Low risk | Zinc was determined by AAS  from stored samples 1–5 years  after collection.  Type 2 DM determined by  self-report and record  linkage to national hospital  discharge registry. |
| Valid measurements of both  exposure and outcome (Zn intake) | N/A |  |
| Selective reporting | Unclear risk | Study protocol identified;  protocol did not include  analysis described  in the paper. |

**Table S4.** Scoring criteria of confounding factors and results of each individual study with models describing zinc intake and CVD outcomes.

| **Study ID** | **Family  History (/0.5)** | **Comorbidities  (/0.5)** | **Lifestyle  Factors (/0.5)** | **Anthropometry  (/0.5)** | **Trans Fat  Intake (/1)** | **Saturated Fat  Intake (/1)** | **Other Dietary  Factors (/0.5)** | **Medication  (/0.5)** | **Total Score (/5)** |
| --- | --- | --- | --- | --- | --- | --- | --- | --- | --- |
| Al-Delaimy et al. 2004 [1] | 0.5 | 0.5 | 0.5 | 0.5 | 1 | 0 | 0.5 | 0.5 | 4 |
| Bates et al. 2011 [2] | 0 | 0 | 0 | 0 | 0 | 0 | 0 | 0 | 0 |
| Lee et al. 2005 [3] | 0.5 | 0 | 0.5 | 0.5 | 1 | 1 | 0.5 | 0.5 | 4.5 |
| Mursu et al. 2011 [6] | 0 | 0.5 | 0.5 | 0.5 | 0 | 1 | 0.5 | 0.5 | 3.5 |
| Otto et al. 2012 [7] | 0 | 0 | 0.5 | 0.5 | 0 | 1 | 0.5 | 0 | 2.5 |

**Table S5.** Scoring criteria of confounding factors and results of each individual study with models describing serum zinc concentration and CVD outcomes.

| **Study ID** | **Total Cholesterol or LDL  Cholesterol Concentration (/1)** | **HDL Cholesterol (/1)** | **Inflammation Biomarker (/1)** | **Total Score (/3)** |
| --- | --- | --- | --- | --- |
| Bates et al. 2011 [2] | 1 | 1 | 1 | 3 |
| Leone et al. 2006 [4] | 1 | 1 | 0 | 2 |
| Marniemi et al. 1998 [5] | 1 | 1 | 0 | 2 |
| Pilz et al. 2009 [9] | 1 | 1 | 1 | 3 |
| Soinio et al. 2007 [10] | 1 | 1 | 0 | 2 |

**Table S6.** Scoring criteria of confounding factors and results of each individual study with models describing zinc intake and Type 2 DM outcomes.

| **Study ID** | **Family  History (/0.5)** | **Comorbidities  (/0.5)** | **Lifestyle  Factors (/0.5)** | **Anthropometry  (/0.5)** | **Saturated Fat  Intake (/1)** | **Dietary Fibre  Intake (/1)** | **Other Dietary  Factors (/0.5)** | **Medication  (/0.5)** | **Total Score (/5)** |
| --- | --- | --- | --- | --- | --- | --- | --- | --- | --- |
| Otto et al. 2012 [7] | 0 | 0 | 0.5 | 0.5 | 0 | 1 | 0.5 | 0 | 2.5 |
| Song et al. 2011 [11] | 0 | 0 | 0.5 | 0.5 | 0 | 0 | 0.5 | 0 | 1.5 |
| Sun et al. 2009 [12] | 0.5 | 0 | 0.5 | 0.5 | 0 | 0 | 0.5 | 0.5 | 2.5 |
| Vashum et al. 2013 [13] | 0 | 0.5 | 0.5 | 0.5 | 0 | 1 | 0.5 | 0.5 | 3.5 |

**Table S7.** Scoring criteria of confounding factors and results of each individual study with models describing serum zinc concentration and Type 2 DM outcomes.

| **Study ID** | **Fasting Glucose or HbA1c (/1)** | **HDL Cholesterol (/0.5)** | **Triglycerides (/0.5)** | **Inflammation Biomarker (/1)** | **Total Score (/3)** |
| --- | --- | --- | --- | --- | --- |
| Soinio et al. 2007 [10] | 1 | 0 | 0 | 1 | 2 |

Reference

1. Al-Delaimy, W.K.; Rimm, E.B.; Willett, W.C.; Stampfer, M.J.; Hu, F.B. Magnesium intake and risk of coronary heart disease among men. *J. Am. Coll. Nutr.* **2004**, *23*, 63–70.
2. Bates, C.J.; Hamer, M.; Mishra, G.D. Redox-modulatory vitamins and minerals that prospectively predict mortality in older British people: The National Diet and Nutrition Survey of people aged 65 years and over. *Br. J. Nutr.* **2011**, *105*, 123–132.
3. Lee, D.-H.; Folsom, A.R.; Jacobs, D.R. Iron, zinc, and alcohol consumption and mortality from cardiovascular diseases: the Iowa Women’s Health Study. *Am. J. Clin. Nutr.* **2005**, *81*, 787–791.
4. Leone, N.; Courbon, D.; Ducimetiere, P.; Zureik, M. Zinc, copper, and magnesium and risks for all-cause, cancer, and cardiovascular mortality. *Epidemiology* **2006**, *17*, 308–314.
5. Marniemi, J.; Järvisalo, J.; Toikka, T.; Räihä, I.; Ahotupa, M.; Sourander, L. Blood vitamins, mineral elements and inflammation markers as risk factors of vascular and non-vascular disease mortality in an elderly population. *Int. J. Epidemiol.* **1998**, *27*, 799–807.
6. Mursu, J.; Robien, K.; Harnack, L.J.; Park, K., Jacobs, D.R., Jr. Dietary supplements and mortality rate in older women. *Arch. Intern. Med.* **2011**, *171*, 1625–1633.
7. Otto, M.C.D.O.; Alonso, A.; Lee, D.; Delclos, G.L.; Bertoni, A.G.; Jiang, R.; Lima, J.A.; Symanski, E.; Jacobs, D.R., Jr.; Nettleton, J.A. Dietary intakes of zinc and heme iron from red meat, but not from other sources, are associated with greater risk of metabolic syndrome and cardiovascular disease. *J. Nutr.* **2012**, *142*, 526–533.
8. Park, J.S.; Xun, P.; Li, J.; Morris, S.J.; Jacobs, D.R.; Liu, K.; He, K. Longitudinal association between toenail zinc levels and the incidence of diabetes among American young adults: The CARDIA Trace Element Study. *Sci. Rep.* **2016**, *6*, doi:10.1038/srep23155.
9. Pilz, S.; Dobnig, H.; Winklhofer-Roob, B.M.; Renner, W.; Seelhorst, U.; Wellnitz, B.; Boehm, B.O.; März, W. Low serum zinc concentrations predict mortality in patients referred to coronary angiography. *Br. J. Nutr.* **2009**, *101*, 1534–1540.
10. Soinio, M.; Marniemi, J.; Laakso, M.; Pyörälä, K.; Lehto, S.; Rönnemaa, T. Serum zinc level and coronary heart disease events in patients with type 2 diabetes. *Diabetes Care* **2007**, *30*, 523–528.
11. Song, Y.; Xu, Q.; Park, Y.; Hollenbeck, A.; Schatzkin, A.; Chen, H. Multivitamins, individual vitamin and mineral supplements, and risk of diabetes among older U.S. adults. *Diabetes Care* **2011**, *34*, 108–114.
12. Sun, Q.; van Dam, R.M.; Willett, W.C.; Hu, F.B. Prospective study of zinc intake and risk of type 2 diabetes in women. *Diabetes Care* **2009**, *32*, 629–634.
13. Vashum, K.P.; McEvoy, M.; Shi, Z.; Milton, A.H.; Islam, M.R.; Sibbritt, D.; Patterson, A.; Byles, J.; Loxton, D.; Attia, J. Is dietary zinc protective for type 2 diabetes? Results from the Australian longitudinal study on women’s health. *BMC Endocr. Disord.* **2013**, *13*, 40.
14. Yary, T.; Virtanen, J.K.; Ruusunen, A.; Tuomainen, T.-P.; Voutilainen, S. Serum zinc and risk of type 2 diabetes incidence in men: the Kuopio Ischaemic Heart Disease Risk Factor study. *J. Trace Elem. Med. Biol.* **2016**, *33*, 120–124.

© 2016 by the authors. Submitted for possible open access publication under the
terms and conditions of the Creative Commons Attribution (CC-BY) license (http://creativecommons.org/licenses/by/4.0/).
